# An Integrated Method for Microwave Absorption and External Thermal Flow Simulation in SAR Antenna Vacuum Thermal Tests

**DOI:** 10.3390/s24123920

**Published:** 2024-06-17

**Authors:** Shangjie Pan, Yuchang Zhang, Chun Liu, Wanqing An, Yu Zhang

**Affiliations:** Beijing Institute of Spacecraft Environment Engineering, Beijing 100094, China; zhangyuchang511@163.com (Y.Z.); liuchun8563@126.com (C.L.); 15811220216@163.com (W.A.); 13811613526@139.com (Y.Z.)

**Keywords:** SAR antenna, low electromagnetic scattering environment, external thermal flow environment, integrated simulation method, vacuum thermal test

## Abstract

The simulation of microwave absorption and external thermal flow is an essential aspect of the vacuum thermal testing process for Synthetic Aperture Radar (SAR) antenna. This paper proposes a novel integrated method for simulating microwave absorption and external thermal flow, specifically designed for vacuum thermal testing. The method employs a non-woven fabric square pyramid assembly as the primary structure to establish a low electromagnetic scattering environment. External heat flow simulation is achieved by arranging carbon fiber heating wires between square cones. Through numerical analysis and experimental tests, the influence of the position of the carbon fiber heating wire on the uniformity of heat flow and reflectivity was revealed. A prototype system is developed based on these findings. The external thermal flow is adjustable in the range of 80–550 W/m^2^, with a uniformity better than 5%. The reflectivity in the L to X microwave frequency band is basically better than −25 dB, and in local frequency bands, it is better than −30 dB. The system has been successfully applied in SAR antenna component and satellite vacuum thermal tests, meeting all ground simulation test requirements and exhibiting significant potential for widespread application.

## 1. Introduction

Synthetic Aperture Radar (SAR) antennas play a vital role in satellite remote sensing due to their characteristics of all-weather, all-day, wide-range, and high-precision observations [1]. The electromagnetic waves emitted by SAR antennas in different bands can penetrate water vapor clouds and image different scattering characteristics of the earth’s surface. In specific frequency bands, subsurface information can even be obtained through the earth’s surface and vegetation [2]. At present, most of the existing SAR antenna frequency bands are concentrated in the L to Ka band [3,4,5]. During satellite operation in orbit, the peak power of microwave radiation emitted by the SAR antenna reaches several kilowatts, while the microwave radiation power received after free space loss and reflection absorption is only watts. Taking China’s Gaofen-3 satellite, whose frequency band is the C-band, as an example, the peak transmit power of the antenna is 2.885 kW [6,7]. When a SAR antenna is tested in a vacuum thermal test, if the microwave radiation signal emitted by the antenna is not absorbed, the high-power microwave radiation signal will be reflected back to the antenna receiving end and cause damage. Therefore, it is necessary to establish a microwave absorption environment during the vacuum thermal test.

Vacuum thermal tests conducted within space environment simulators are to validate the functionality and performance of satellites and onboard products in space conditions [8]. The external heat flow in space conditions mainly consists of solar radiation, earth radiation, and reflection. SAR antennas are mainly used for earth remote sensing observations, and their radiated heat flux mainly comes from the earth. The earth’s surface absorbs solar energy, converts it into heat energy, and then radiates it to space in the form of long waves. Due to the large thermal inertia of the earth, the temperature remains basically unchanged, and the heat flow radiated to space changes very little, about 234 W/m^2^ [9]. In vacuum thermal testing, devices such as infrared heating cages and infrared light arrays are often used to simulate external heat flow environments [10,11]. These devices can easily meet the external heat flow requirements of the antenna vacuum thermal test, but due to their high reflectivity to microwaves, they cannot meet the microwave absorption environment required for SAR antenna testing.

Absorbing materials can convert the energy of electromagnetic waves projected onto their surfaces into heat or other forms of energy through dielectric loss [12]. The use of absorbing materials to establish a microwave absorption environment has been widely applied in military and civilian fields [13,14]. In the context of SAR antenna vacuum thermal tests, the United States, Japan, Italy, and others have utilized Eccosorb SF absorbing materials for building microwave absorption environments [15]. The limitation of this method is that it can only provide a microwave absorption environment, but it cannot simulate the radiation heat flux experienced by the SAR antenna when it is in orbit. In China, silicon carbide square pyramid components are mainly used to construct a microwave absorption environment, and the temperature of the square pyramid components is controlled by heating elements to achieve external heat flow simulation [16]. However, due to limitations in the processing technology of silicon carbide cone components, their structural dimensions are generally small, resulting in poor absorption of low-frequency microwaves. In addition, this method needs to indirectly simulate the external heat flow by heating the silicon carbide cone component. Due to the large heat capacity of the silicon carbide cone component, there are defects such as slow heat flow adjustment and poor uniformity, which increases the time and cost required for vacuum thermal experiments.

In response to these issues, this paper proposes a method for simulating microwave absorption and external heat flow integration to meet the thermal vacuum test requirements of antennas with transmission frequencies above 1 GHz, achieving a composite background with a low electromagnetic scattering environment and adjustable heat flow. The feasibility and performance of the method are verified through numerical analysis and experiments, and a prototype system is designed. The system is applied to the vacuum thermal tests of a satellite SAR antenna. The data indicate that throughout the entire vacuum thermal test process, the device provides a low electromagnetic scattering environment for satellite SAR under various working conditions. The simulated external thermal flow is adjustable in the range of 80–550 W/m^2^, with a uniformity better than 5% and a thermal flow variation rate not less than 100 W/m^2^·h. This method significantly reduces the high and low-temperature cycling time in vacuum thermal tests, and all indicators meet the requirements for simulating the microwave absorption and external thermal flow environment of the SAR satellite.

## 2. Integrated Simulation System Design

### 2.1. Microwave Absorption Subsystem

**Figure 1:**
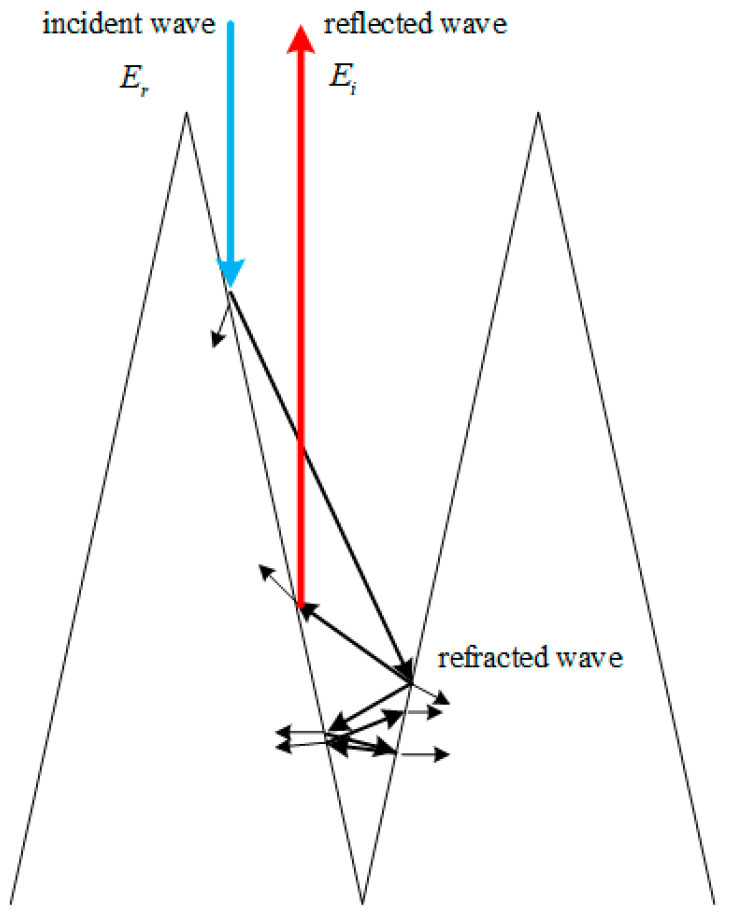
Schematic diagram of electromagnetic wave reflection and refraction between cones.

The microwave reflectivity is a key indicator for evaluating absorption performance. As shown in Figure 1, using a uniform planar wave incident along the normal direction of the pyramidal absorbing material, measuring the amplitudes of the incident and reflected waves separately, the reflection loss R in decibels is obtained:

(1) $$R\left(dB\right)=20\log_{10}\left(\frac{E_{r}}{E_{i}}\right),$$

where $E_{r}$ is the amplitude of the incident wave, measured in V/m, and $E_{i}$ is the amplitude of the reflected wave, also measured in V/m. The higher the reflection coefficient, the better the microwave absorption performance.

According to the Friis transmission formula, when the antenna is at a distance d away from the absorbing material and the frequency of the emitted microwave is f, the free space transmission loss $L_{0}$ can be calculated by the following formula.

(2) $$L_{0}(\mathrm{dB})=92.4+20\log_{10}(2d)+20\log_{10}f$$

where the unit of d is km, and the unit of f is GHz.

It can be known from Formula (2) that space transmission loss is positively related to microwave transmission distance and frequency. However, in a vacuum thermal test of SAR antennas, the distance between the antenna and the absorbing material is limited by the size of the space environment simulator. Taking an extreme case analysis, when the minimum distance between the antenna and the absorbing material is 200 mm for microwaves with a frequency above 1 GHz, calculated from Formula (2), it can be seen that the minimum free space transmission loss is 24.44 dB.

Furthermore, it is assumed that the sum of free space loss $L_{0}$ and reflection loss R is greater than 50 dB. Even if a microwave with a power of 10,000 W is transmitted and absorbed by the material, the microwave power received is only 0.1 W, which is completely acceptable for the vacuum thermal test of the SAR antenna. Therefore, in the vacuum thermal test of the SAR antenna, an environment where the reflection loss established by the absorbing material is better than 25 dB can meet the antenna test requirements.

The non-woven fabric absorbing material is a hollow absorbing body made of flame-retardant, carbon black-impregnated, and temperature-resistant non-woven fabric as the base material. It has the characteristics of a wide operating frequency range and good absorbing performance. The non-woven fabric square pyramid assembly is a structure in which non-woven fabric is laminated into a layered square cone through compression, cutting, and other processes. The square-cone shape causes incident electromagnetic waves to undergo multiple reflections between adjacent cones, accompanied by significant refraction, as depicted in Figure 1. The layered structure enhances the interaction of incoming electromagnetic waves with the material, resulting in increased absorption [17]. The non-woven fabric square pyramid assembly exhibits characteristics such as strong absorption ability and low cost, making it widely used in EMC chambers. It also meets the requirements for vacuum thermal tests in terms of material volatility and high-low temperature tolerance. Therefore, it is a good choice to use a non-woven fabric square pyramid assembly as the absorbing material of the integrated simulation system.

When establishing a microwave absorption environment using square cone components, the height of a single cone should generally be one wavelength at the lowest frequency [18]. To ensure the integrated simulation system has microwave absorption capabilities at 1 GHz and above, the chosen height of the non-woven fabric square pyramid assembly is set to 320 mm. The non-woven fabric absorbing material selected in this paper has a power tolerance of 5 kW/m^2^, the reflectivity of vertically incident microwaves from 1 to 40 GHz is less than −30 dB, and its absorbing performance does not change when its temperature is not higher than 150 °C. When a non-woven fabric square pyramid assembly absorbs microwaves and converts them into internal energy, causing its own temperature to rise, it can dissipate heat through thermal radiation. The cold black environment (temperature below 100 K) established by the space environment simulator can effectively reduce the temperature of the absorbing material.

### 2.2. External Thermal Flow Simulation Subsystem

In vacuum thermal tests, the vacuum degree should be better than $6.65\times 10^{-3}$ Pa, and radiative heat transfer is the main way for external heat flow simulation devices to heat satellites. During the test, the temperature adjustment rate of satellites mainly depends on the change in radiative heat flux of the external thermal flow simulation device. The change in radiative heat flux ∆Q is defined as:

(3) $$∆Q=\sigma \varepsilon X{\left(T_{i}+\Delta T\right)}^{4}-X\sigma \varepsilon {T_{i}}^{4},$$

where σ is the Stefan–Boltzmann constant, with a value of $5.67\times 10^{-8}W/(m^{2}\cdot K^{4})$; ε is the emissivity of the external thermal flow simulation device, ranging from 0 to 1; X is the angle factor between the external thermal flow simulation device and the satellite, which is only related to their relative position and size; $T_{i}$ is the temperature of the external thermal flow simulation device at the current moment (in Kelvin); and ΔT is the temperature change at the current moment compared to the previous moment (in Kelvin). 

As indicated by the equation, rapidly adjusting the temperature of the external thermal flow simulation device can enhance the temperature adjustment rate of the SAR antenna. Therefore, the external thermal flow simulation device is installed between the SAR antenna and the non-woven fabric square pyramid assembly. The temperature change rate of the external thermal flow simulation device is rapidly adjusted by controlling its current, thereby achieving the goal of improving the SAR antenna’s temperature adjustment rate.

The uniformity of radiative heat flow is a crucial indicator for evaluating external thermal flow simulation, directly affecting the effectiveness of spacecraft vacuum thermal tests, with an error generally required to be less than 10%. The radiative heat flow uniformity error $Q_{u}$ is defined as:

(4) $$Q_{u}=\pm \frac{E_{max}-E_{min}}{E_{max}+E_{min}}\times 100\%,$$

where $Q_{max}$ is the maximum heat flow reached in the radiation heating area (in W/m^2^), and $Q_{min}$ is the minimum heat flow reached in the radiation heating area (in W/m^2^).

The uniformity of radiative heat flow is closely related to factors such as the type of external thermal flow simulation device and the relative position with the satellite product. 

Choosing a thermal flow simulation device with high emissivity, low deformation, and uniform electrical heating temperature, positioned consistently with the satellite product and heating in the forward direction, can effectively enhance the uniformity of radiative heat flow.

Carbon fiber can be heated to extremely high temperatures, with an emissivity close to 1. It has the characteristics of high electrothermal radiation efficiency, uniform electrothermal radiation, high strength, and minimal deformation under temperature changes [19,20]. This meets the requirements for simulating external thermal flow in vacuum alternating hot and cold environments. In addition, its mass loss rate under vacuum is less than 0.09% (measured at 200 °C), and it can work reliably for a long time. While carbon fiber is a microwave loss medium, its absorption performance is influenced by the direction of the incident electric field, which requires a reasonable layout [21].

Based on the above theoretical analysis, a design proposal for an integrated simulation system for microwave absorption and external thermal flow is presented: a non-woven fabric square pyramid assembly with a single cone height of 320 mm and a width of 62.5 mm is used for microwave absorption. For the external thermal flow simulation device, carbon fiber heating wires with a diameter of 1 mm and a resistivity of 33 Ω/m are used, uniformly arranged in the gaps between the cones of the non-woven fabric square pyramid assembly.

## 3. Performance of Integrated Simulation System Based on Numerical Analysis

### 3.1. Microwave Absorption Performance Numerical Analysis

Microwave absorption performance numerical analysis is conducted using Ansys Electronics Desktop 2019 R3. A model of 4 × 4 non-woven fabric square cones was built for numerical analysis, with the dimensions of each cone as described above and the cones point in the +Z direction. The surface of the model is set to layer impedance boundary condition, the relative permittivity of the layer material equals 6, its relative permeability is 1, its dielectric loss tangent is 0.5, and its magnetic loss tangent is 0.5. The wavelength of a microwave is inversely proportional to the frequency, and the absorption effect of conical absorbing materials is worse for low-frequency microwaves [19]. In order to obtain the performance of the integrated simulation system under the harshest conditions, the excitation source is a uniform plane wave with a frequency of 1 GHz, incident from the top of the cone assembly along the -Z direction. The polarization direction is along the Y-axis, and the incident field intensity amplitude is set to 1 V/m.

**Figure 2:**
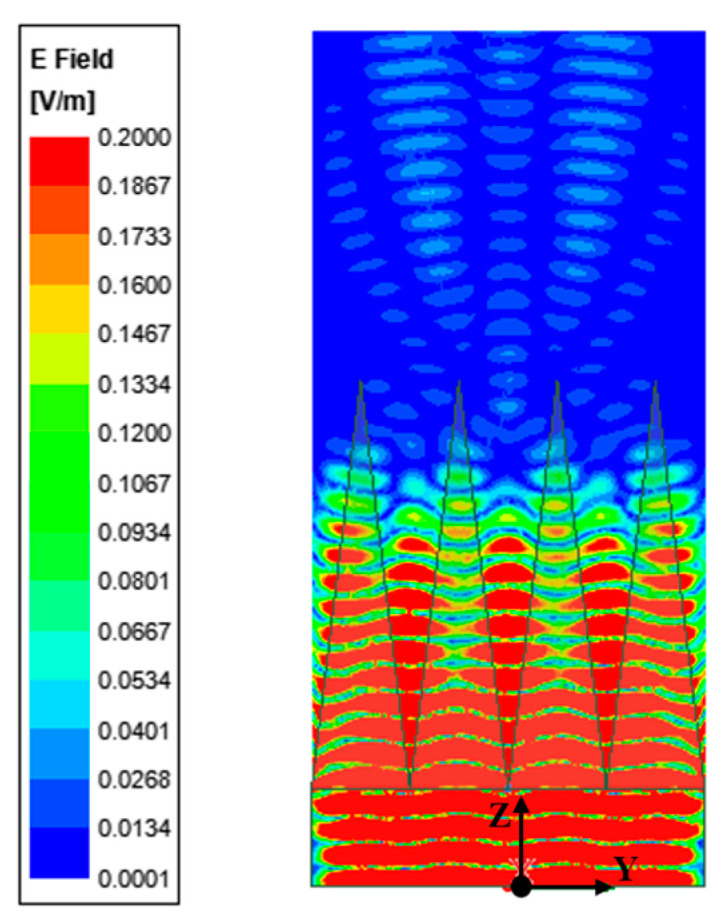
Field intensity distribution near the cone assembly.

**Figure 3:**
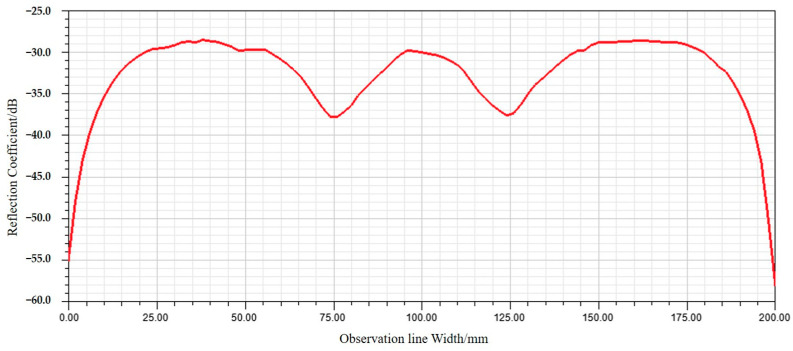
Reflection coefficient of the cone assembly on the observation line.

Under these conditions, the distribution of the reflected field intensity is obtained, as shown in Figure 2; most microwave energy is absorbed by non-woven fabric square pyramid assembly. Additionally, a 200 mm long observation line was drawn along the Y-axis in the center of the plane, which was located 200 mm above the apex of the non-woven square cone and was the closest installation position to the SAR antenna in the vacuum thermal test. The reflection coefficient obtained from the observation line is shown in Figure 3, and the results show that the reflectance around the line fluctuates. The reflection coefficient is about −30 dB, which is basically consistent with the actual performance of the selected non-woven fabric assembly. Therefore, the model can effectively replace the actual absorbing material for subsequent numerical analysis.

Further, consider the need for carbon fiber heating wires to be evenly distributed between the cones of the non-woven fabric square pyramid assembly to achieve external heat flow simulation. Set the heating wire 100 mm below the top of the cone. The carbon fiber heating wires are simplified to a cylindrical style with a diameter of 1 mm, a relative permittivity of 8, and a conductivity of 1000 S/m. Since its temperature coefficient is about −0.05%/℃, the change in resistance decreasing with increasing temperature is negligible. Therefore, the conductivity is set to a constant.

**Figure 4:**
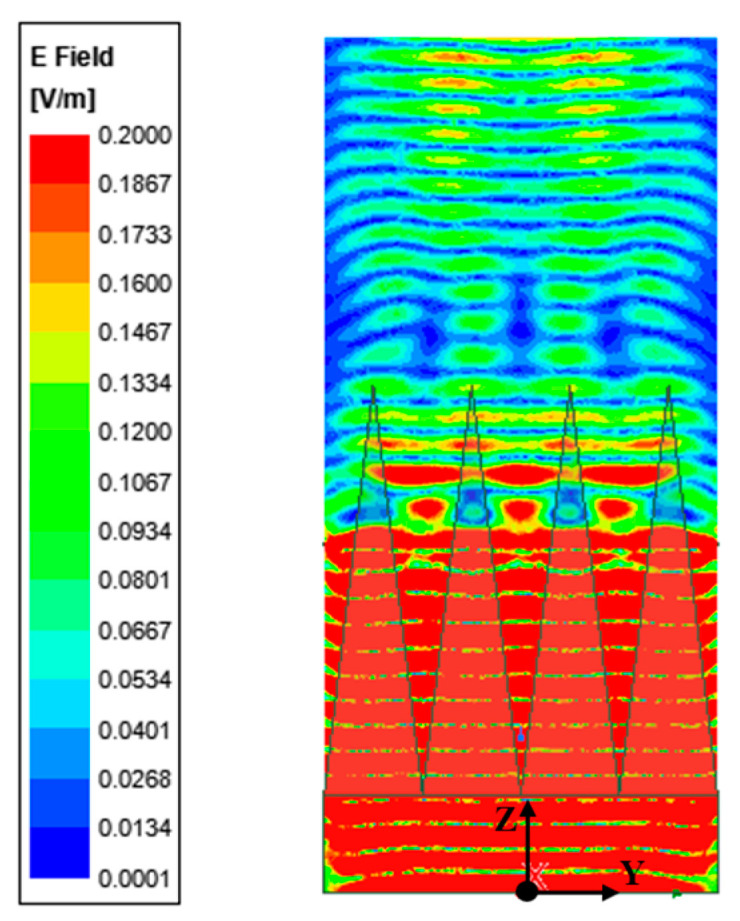
Reflected field intensity distribution with parallel wiring to polarization direction.

**Figure 5:**
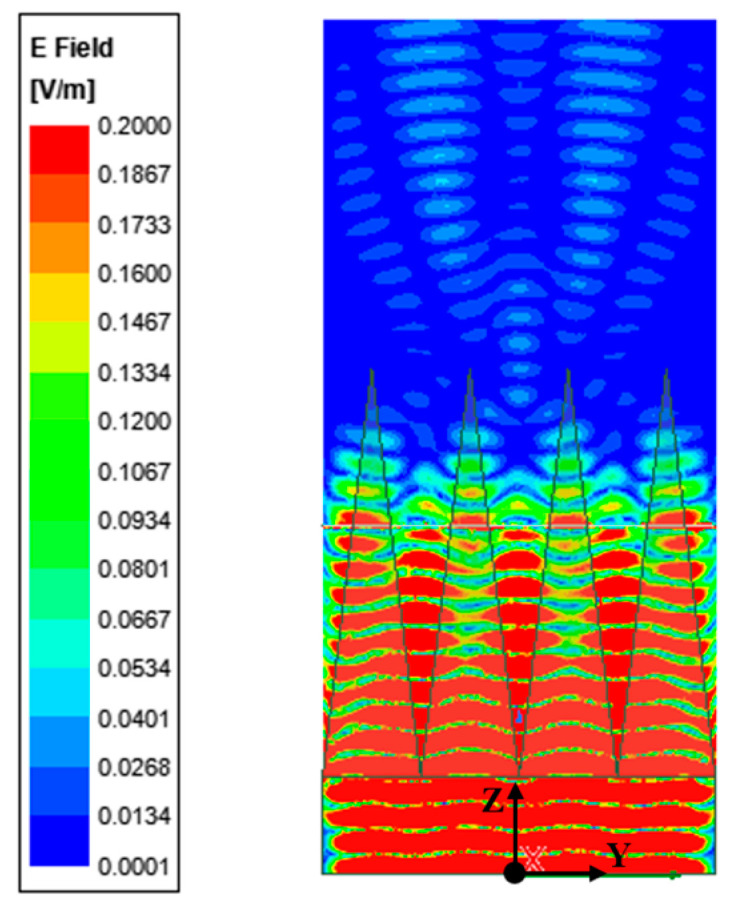
Reflected field intensity distribution with perpendicular wiring to polarization direction.

**Figure 6:**
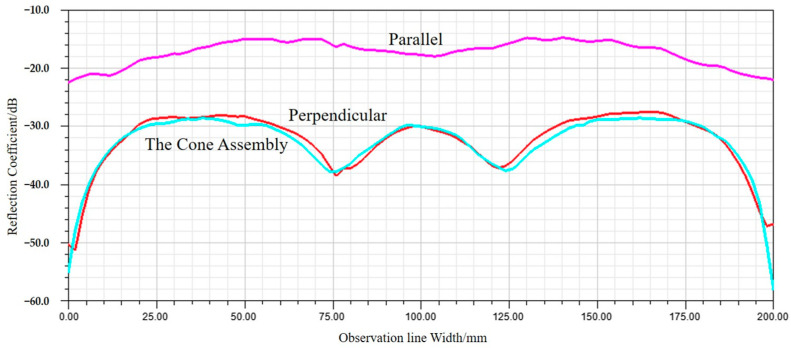
Reflection coefficients on the observation line for different wiring directions.

With other conditions unchanged, numerical analysis is performed in two situations: when the heating wires are parallel and perpendicular to the polarization direction of the antenna. The distributions of the reflected field intensity for different wiring configurations are shown in Figure 4 and Figure 5. The reflection coefficients for different wiring configurations are shown in Figure 6.

From the numerical analysis results, it can be concluded that when the direction of the heating wires is parallel to the electric field polarization direction, strong induced currents on the heating wires lead to significant scattering, resulting in a reduction in the reflection coefficient to around −19 dB. When the direction of the heating wires is perpendicular to the electric field polarization direction, the scattering is reduced, and the reflection coefficient is maintained at around −30 dB. Obviously, the direction of the heating wire is perpendicular to the direction of electric field polarization, which is conducive to obtaining a better microwave absorption environment.

**Figure 7:**
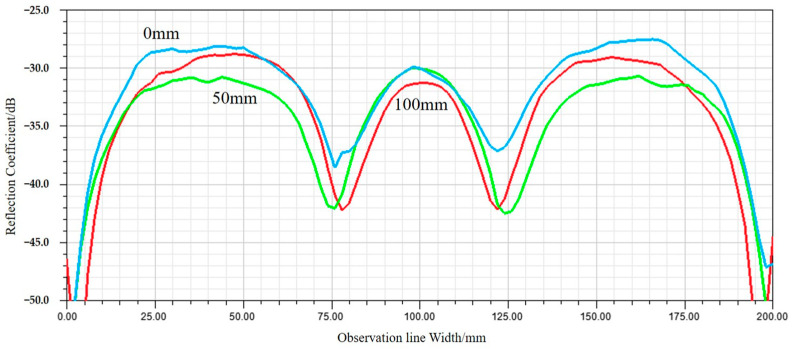
Reflection coefficients on the observation line for different wiring depths.

Furthermore, a numerical analysis is performed on the relationship between the depth position of the heating wire and the reflectivity. Under the condition where the heating wire routing direction is perpendicular to the electric field polarization direction, models are constructed with the heating wires positioned 0 mm, 50 mm, and 100 mm below the top of the cones. When other conditions are unchanged, the reflection coefficients at different wiring depths are shown in Figure 7.

There are certain differences in the reflection coefficients at the three heating wire depth positions. When the heating wires are wired at a depth of 0 mm, the proportion of electromagnetic waves scattered by the heating wires back to the incident direction is relatively high, resulting in a higher reflection coefficient and poorer absorption performance. However, if the heating wires are wired at depths of 50 mm and 100 mm, the proportion of the scattered field by the heating wires returning to the incident direction is smaller, and most of it returns to the surface of the non-woven fabric square pyramid assembly, leading to better absorption performance and meeting the requirements for a low electromagnetic scattering environment. Therefore, the wiring depth of the heating wires should generally be better than 50 mm.

### 3.2. Numerical Analysis of External Thermal Flow Simulation Uniformity

Simulation performance analysis of external thermal flow is carried out using the Monte Carlo method. An assembly of 8 × 8 non-woven fabric square cones is selected. Carbon fiber heating wires are uniformly distributed 50 mm from the top of the cones. The carbon fiber heating wires are simplified to a cylindrical style with a diameter of 1 mm, a length of 500 mm, and a resistivity of 0.01 Ω/mm, calculated for cylindrical heating.

The heated surface area is 500 mm×500 mm, located 200 mm from the top of the cones of the non-woven fabric square pyramid assembly. A rectangular coordinate system is established with the lower-left corner of the heated plane as the origin. The direction of the heating wire is the X-axis, and the distance direction between the heated surface and the non-woven fabric square pyramid assembly is the Z-axis. Assuming that a particle is emitted from the nth carbon fiber heating filament, particles are emitted with an apex angle φ and an azimuthal angle ω, modeled by the probability distribution:

(5) φ=2πa,

(6) $$\omega =\mathrm{arc}\sin\sqrt{\beta },$$

where a and β are random numbers between 0 and 1.

By calculating the geometric relationships, the coordinates of the intersection point between the particle and the heated surface can be obtained, and it can be determined whether the particle falls on the heated surface. When a sufficient number of particles are emitted, the number of particles falling in different regions of the heated plane can be used to characterize the uniformity of radiative heat flow.

**Figure 8:**
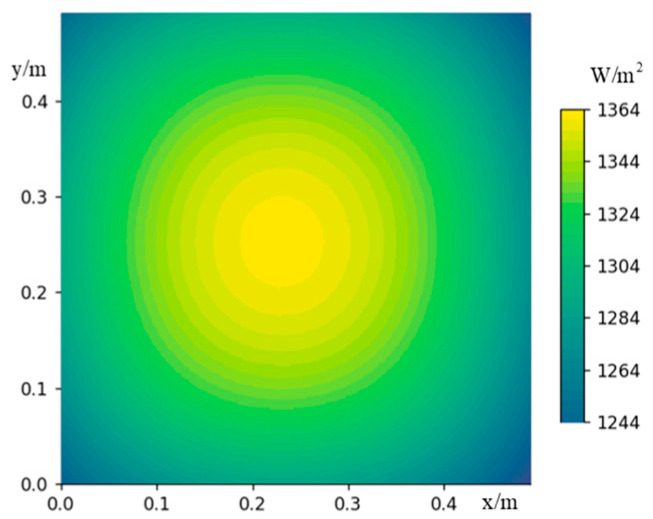
Numerical analysis results of radiative heat flow on the heated surface.

Based on this principle, numerical analysis calculations are performed. The heating wires are evenly distributed in the non-woven fabric square pyramid assembly, with a length of 500 mm. The resistivity is 33 Ω/m. When a current of I = 2 A is applied to all carbon fiber heating wires, it can be known from Ohm’s law that the total heat energy generated by the heating wires is 528 W. The heated surface is heated in a thermal equilibrium state, and the theoretical heat flux is 2112 W/m^2^. Combined with the particle distribution probabilities in different areas of the heated surface, the numerical analysis results of the radiant heat flow on the heated surface are shown in Figure 8, and the uniformity error of radiative heat flow $Q_{u}=4.6\%$.

## 4. Experimental Analysis of Integrated Simulation System

### 4.1. Microwave Absorption Experiment and Performance Analysis

**Figure 9:**
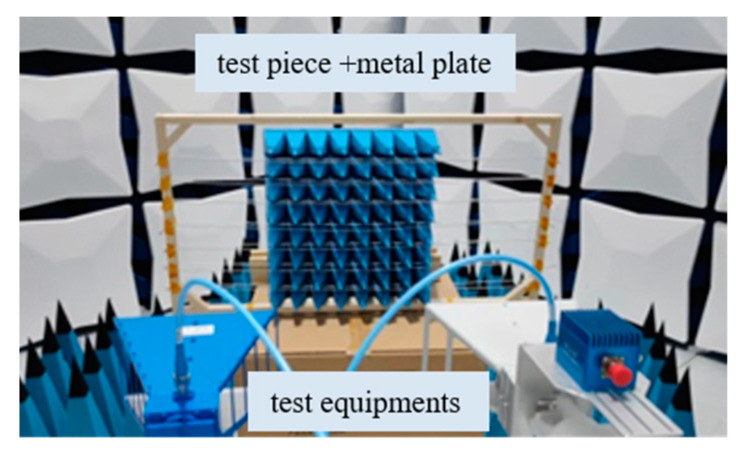
Experimental verification system.

The Radar Cross Section (RCS) testing method is a common method for testing the reflectivity of microwave absorption materials. The test piece is subjected to reflectivity testing in a compact field using a vector network analyzer, signal transceiver equipment, etc., as shown in Figure 9. According to the above numerical analysis results, the test piece includes an assembly of 8 × 8 non-woven fabric square cones, with heating wires uniformly distributed 50 mm from the top of the cones. The test steps are as follows:

Step 1: Use a standard metal ball for calibration, and then measure the theoretical RCS value $\Gamma_{m}(\mathrm{dB})$ of the metal plate that is consistent with the size of the test piece;

Step 2: Keep the position of the metal plate unchanged, place the test piece in front of the metal plate, and measure the RCS value $\Gamma_{\mathrm{RAM}}(\mathrm{dB})$ of the test piece in the same way.

Step 3: Make a difference between the RCS value $\Gamma_{\mathrm{RAM}}(\mathrm{dB})$ measured on the test piece at each frequency and the theoretical RCS value $\Gamma_{m}(\mathrm{dB})$ of the metal plate, and the reflectivity of the non-woven fabric square cones with heating wires at each frequency can be obtained.

**Figure 10:**
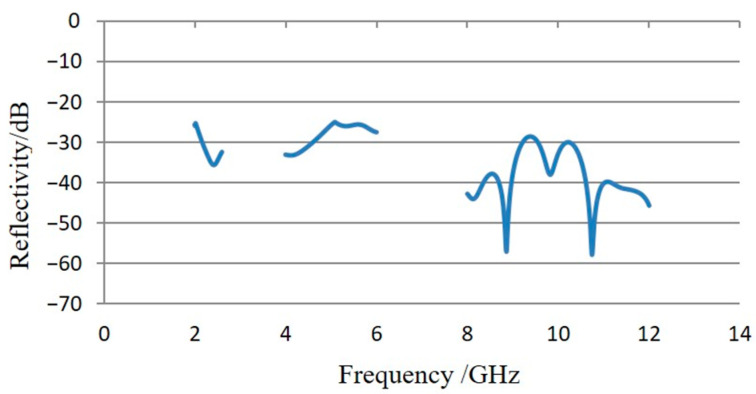
Reflectivity test results using RCS method.

Experiments were conducted with frequency sweeping in three continuous microwave frequency bands: 1–2.6 GHz, 4–6 GHz, and 8–12 GHz. The reflectivity test results are shown in Figure 10. According to the test results, the reflectivity of the test piece is better than −25 dB for microwave signals in the 1–2.6 GHz and 4–6 GHz frequency bands and better than −30 dB for the 8–12 GHz frequency band. Overall, it exhibits good absorption performance, meeting the requirements for low electromagnetic scattering environments in the vacuum thermal test of SAR antennas.

### 4.2. External Thermal Flow Simulation Experiment and Performance Analysis

Since the absorbing material absorbs microwaves and converts the microwave energy into internal energy, the overall temperature of the material increases, which is beneficial to increasing the simulated external thermal flow and improving the uniformity of the heat flow. Therefore, this paper simplifies the experiment and tests the heat flow simulation capability without microwave incidence. 

**Figure 11:**
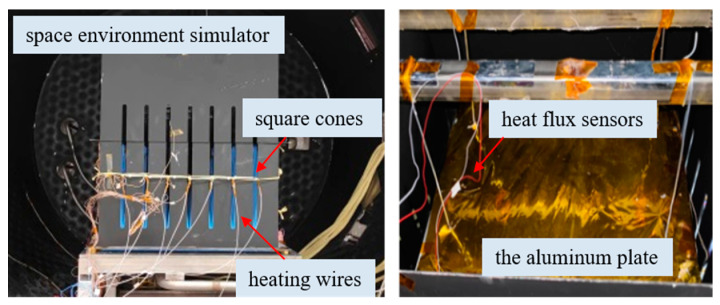
External thermal flow simulation experiment setup.

As shown in Figure 11, uniformity testing of external thermal flow simulation was conducted on a 2 m diameter space environment simulator. The test piece includes an assembly of 8×8 non-woven fabric square cones, with heating wires uniformly distributed 50 mm from the top of the cones. The heated surface uses a 5 mm thick aluminum plate, and the back of the aluminum plate is covered with 20 units of multi-layer insulation components. Six heat flux sensors are attached to the side facing the carbon fiber heating wires as heat flux measurement points. This sensor is an adiabatic heat flux sensor commonly used in spacecraft vacuum thermal tests, with a measurement range of 50–2100 W/m^2^ and a measurement error of less than 5%. Sensors 3 and 4 are located in the center of the aluminum plate, and the remaining 4 are located at the midpoint of the four sides, 15 mm from the edge.

**Figure 12:**
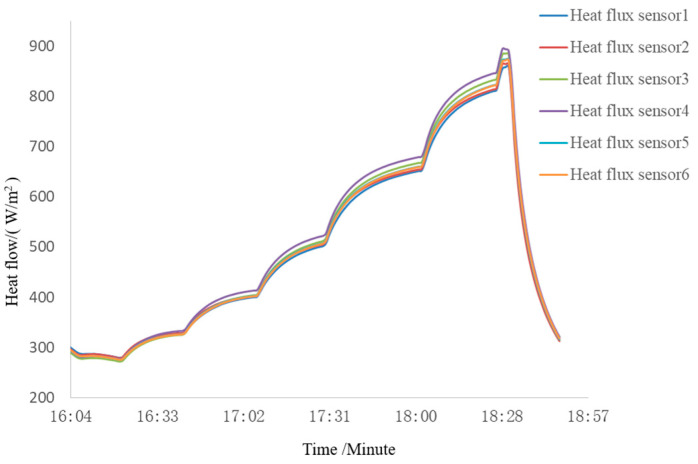
Experimental results of external thermal flow simulation capability.

The background temperature during the experiment was below 100 K, and the vacuum degree was better than $6.65\times 10^{-3}$ Pa. The aluminum plate was tested under seven predetermined conditions. Each condition transitioned to the next after a temperature equilibrium of 3 min, with the temperature fluctuation criterion being: temperature fluctuation value does not exceed ±0.5°C/min. The radiative heat flux measurement curves of the heat flux sensors on the aluminum plate during the experiment are shown in Figure 12. The change trends of radiative heat flux were basically consistent during the experiment and the radiative heat flux uniformity error $Q_{u}<5\%$, consistent with the simulation results. When a current of I = 1.8 A is applied to all carbon fiber heating wires, the maximum heat flux is close to 900 W/m^2^, which meets the experimental requirements of the antenna thermal vacuum test.

## 5. Integrated Simulation System Design and Application Case

For a satellite SAR antenna with an effective aperture of 3.9 m (length) × 1.1 m (width) and a frequency of 9.6 GHz (X-band), which is a flat-panel structure consisting of three independent panels, an integrated simulation system was designed to provide microwave absorption and external thermal flow simulation capabilities.

**Figure 13:**
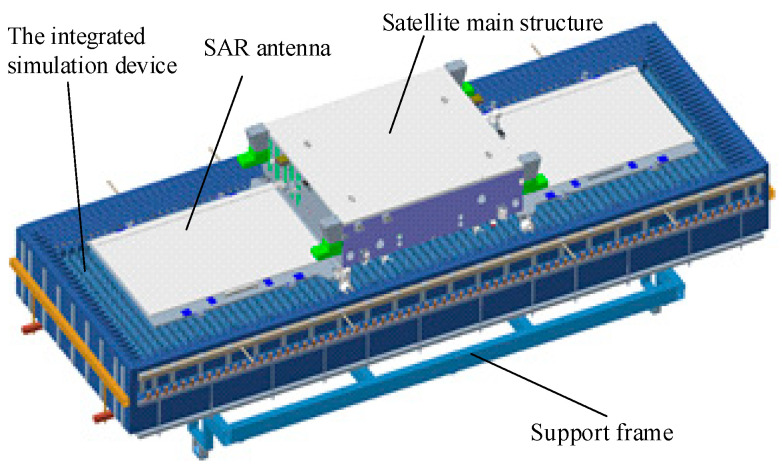
Relative installation position relationship between the satellite and the integrated simulation system.

As shown in Figure 13, the integrated simulation system is a single-sided open box structure, including a support frame, non-woven fabric absorbing materials, and carbon fiber heating wires. The support frame adopts a hollow metal truss structure, and non-woven fabric square cones are fixed inside the support frame using screws, covering the entire inner wall of the device. The non-woven fabric square pyramid assembly has a single cone height of 320 mm and a bottom length and width of 62.5 mm. Carbon fiber heating wires are evenly distributed along the gaps between the cones, 50 mm below the cone apex, with the wiring direction perpendicular to the polarization direction of the SAR antenna. The carbon fiber heating wires extend out of the support frame, and their metal extension wires are welded and connected to an externally controlled DC power supply on the space environment simulator.

During the vacuum thermal test, the integrated simulation system is horizontally mounted on the mounting bracket of the space environment simulator. The SAR antenna is installed on the open side of the integrated simulation device, with the antenna facing the bottom of the device, 200 mm from the top of the non-woven fabric square pyramid assembly. The relative installation position relationship between the satellite and the integrated simulation system is shown in Figure 13.

**Figure 14:**
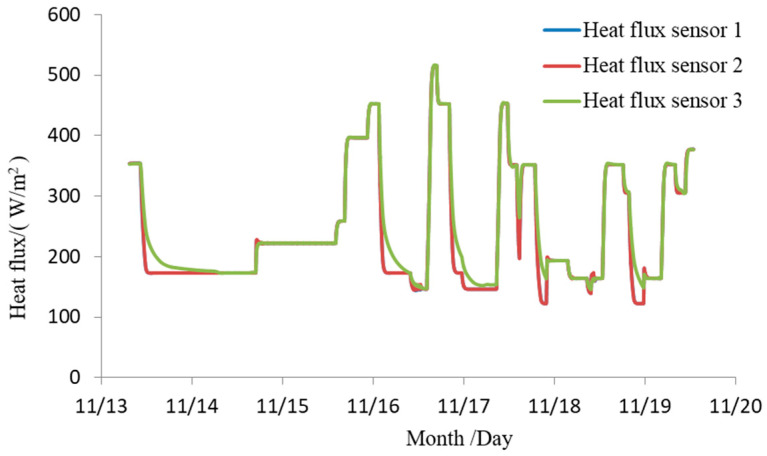
Measured results of external thermal flow in the integrated simulation system.

**Figure 15:**
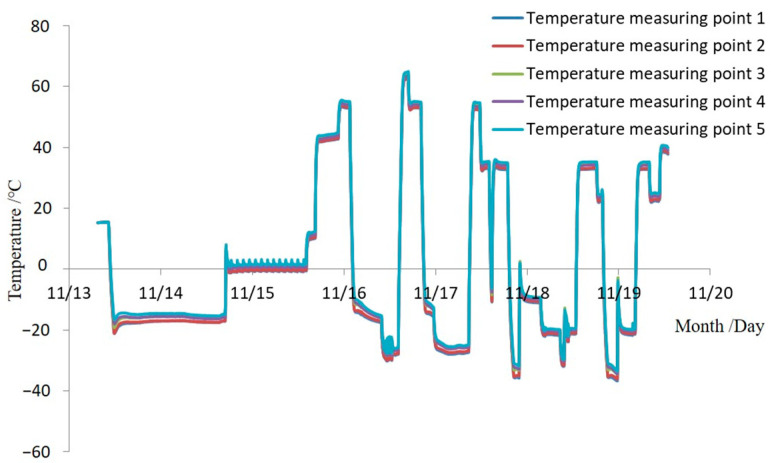
Measured results of absorbing material temperature in the integrated simulation system.

Three heat flux sensors and five thermocouples are installed on the face of the SAR antenna. During the satellites’ vacuum thermal test, closed-loop control is performed based on the temperature and heat flux collected by the black piece heat flux sensors and thermocouples, with a collection period of 1 min. The measured results are shown in Figure 14 and Figure 15. The results indicate that the integrated simulation system effectively absorbs the microwaves emitted by the SAR antenna under various test conditions during the vacuum thermal test. It provides an external thermal flow simulation thermal boundary with a range of 80–550 W/m^2^ and a heat flow uniformity better than 5%. The maximum heat flow change rate can reach 150 W/m^2^·h, which helps to reduce the high and low-temperature cycle time of vacuum thermal testing. The temperature of the absorbing material remains below 150 °C throughout the microwave absorption and external thermal flow variation process. The system meets the requirements for microwave absorption and external thermal flow simulation in the vacuum thermal test of SAR satellites, exhibiting advantages such as low reflectivity, good heat flow uniformity, and fast heat flow adjustment.

## 6. Conclusions

To meet the needs of microwave absorption and external heat flow simulation in vacuum thermal tests of SAR antennas, this paper proposes and designs an integrated simulation system assembled with non-woven fabric square pyramid assembly and carbon fiber heating wires. The contributions of this paper can be summarized as follows:

First, based on the theoretical analysis of microwave absorption of pyramidal materials and radiation heat transfer, the design of an integrated simulation device was carried out. Combining the free space transmission loss and the actual test status of the SAR antenna vacuum thermal test, the specifications of the absorbing material and heating wire were determined, and the reflection loss of the integrated simulation device needed to be no less than 25 dB.

Second, through numerical analysis, the microwave absorption performance and external heat flow simulation capabilities of the integrated device were obtained. The analysis results show the following: (1) The direction of the heating wire should be perpendicular to the direction of electric field polarization, and the depth should be better than 50 mm, which is conducive to obtaining a better microwave absorption environment. (2) When the SAR antenna is 200 mm away from the absorbing material, the integrated device can establish a microwave absorption environment with a reflection coefficient of less than −25 dB. (3) The integrated device has good heat flow simulation capability, and the uniformity error of heat flow is less than 5%.

Third, reflectivity and external heat flow simulation experiments were conducted. The reflectivity test results show that the reflectivity of the integrated device is better than −25 dB for microwave signals in the 1–2.6 GHz and 4–6 GHz frequency bands and better than −30 dB for the 8–12 GHz frequency band. Additionally, the integrated device can provide a maximum heat flux of nearly 900 W/m^2^ with a uniformity of less than 5%. These results prove that the integrated device meets the experimental requirements of the antenna thermal vacuum test.

Based on the above results, an integrated simulation system is designed and applied to satellite SAR antenna thermal vacuum testing. The designed system is characterized by low development cost, easy assembly, and a short development cycle. Each carbon fiber heating wire is independently controlled using a high-precision programmable DC power supply. Based on the requirements of external heat flow simulation of SAR antenna, closed-loop simulation control is adopted, which has the advantages of high simulation accuracy, good uniformity, and fast adjustment speed. The external thermal flow is adjustable in the range of 80–550 W/m^2^, with a thermal flow variation rate not less than 100 W/m^2^·h. The reflectivity in the L to X microwave frequency band is basically better than −25 dB. The device has been successfully used in vacuum thermal tests of X-band SAR antenna components and satellites, and the test results meet the specified requirements.

## Data Availability

The raw data supporting the conclusions of this article will be made available by the authors upon request.
